# In Vitro Susceptibility and Synergistic Effect of Bismuth Against *Helicobacter pylori*

**DOI:** 10.3390/antibiotics13111004

**Published:** 2024-10-25

**Authors:** Jieun Woo, Chang Seok Bang, Jae Jun Lee, Ji Yong Ahn, Jung Mogg Kim, Hwoon-Yong Jung, Eun Jeong Gong

**Affiliations:** 1 Institute of New Frontier Research, Hallym University College of Medicine, Chuncheon 24252, Gangwon-do, Republic of Korea; wje2256@naver.com (J.W.); csbang@hallym.ac.kr (C.S.B.); iloveu59@hallym.ac.kr (J.J.L.); 2 Department of Internal Medicine, Hallym University College of Medicine, Chuncheon 24253, Gangwon-do, Republic of Korea; 3 Department of Anesthesiology and Pain Medicine, Hallym University College of Medicine, Chuncheon 24253, Gangwon-do, Republic of Korea; 4 Department of Gastroenterology, Asan Medical Center, University of Ulsan College of Medicine, Seoul 05505, Republic of Korea; ahnjy@amc.seoul.kr (J.Y.A.); hyjung@amc.seoul.kr (H.-Y.J.); 5 Department of Microbiology, Hanyang University College of Medicine, Seoul 04763, Republic of Korea; jungmogg@hanyang.ac.kr

**Keywords:** anti-bacterial agents, antimicrobial stewardship, bismuth, bismuth tripotassium dicitrate, drug interactions, *Helicobacter pylori*

## Abstract

**Background/objectives:** Bismuth is commonly used in *Helicobacter pylori* (*H. pylori*) eradication therapy. However, few studies have examined the in vitro susceptibility of *H. pylori* to bismuth. Moreover, the exact mechanism of action of bismuth on *H. pylori* remains unclear. The aim of this study was to identify the anti-bacterial effect of bismuth as well as to evaluate potential synergistic effects between bismuth and various antibiotics. **Methods:** The minimum inhibitory concentrations (MICs) of three bismuth preparations, bismuth subsalicylate, bismuth potassium citrate, and colloidal bismuth subcitrate (CBS, De-Nol) were determined for *H. pylori* strains using the agar dilution technique. Agar plates of varying pH values from 5.0 to 8.0 were used to investigate whether acidity influences the anti-bacterial effect of bismuth. A checkerboard assay was performed to assess the synergism between CBS and antibiotics (amoxicillin, clarithromycin, and metronidazole). **Results:** Twelve *H. pylori* strains, including three reference strains (*H. pylori* 26695, J99, and ATCC 43504), and nine clinically isolated strains were tested. The MICs for bismuth subsalicylate, bismuth potassium citrate, and CBS ranged from 4 to 32 μg/mL, 2 to 16 μg/mL, and 1 to 8 μg/mL, respectively. The bismuth MICs for the reference strains were similar at pH 5–8. In the checkerboard assay, no interactions between CBS and any of the antibiotics were observed in the reference *H. pylori* strains. **Conclusions:** Bismuth showed in vitro susceptibility against *H. pylori*. The enhanced eradication efficacy of bismuth-containing regimens appears to be due to mechanisms other than direct synergy with antibiotics.

## 1. Introduction

*Helicobacter pylori* (*H. pylori*) is a gram-negative organism that colonizes an acidic niche in the human stomach [1]. *H. pylori* infection is well-known to be related to the development of chronic gastritis, peptic ulcer diseases, gastric mucosa-associated lymphoid tissue lymphoma, and gastric cancer [2]. In addition, *H. pylori* infection has recently been reported to be associated with extragastric diseases such as iron deficiency anemia, cardiovascular diseases, and Parkinson’s disease [3]. *H. pylori* eradication therapy may improve or relieve the associated pathology and has the potential to prevent gastric cancer [3,4,5].

Despite the long history of *H. pylori* infection since its discovery, *H. pylori* eradication therapy remains suboptimal. The regimens for *H. pylori* eradication consist of acid-suppressive agents in combination with at least two antibiotics such as amoxicillin, clarithromycin, metronidazole, or tetracycline and/or bismuth compounds [6,7]. However, successful eradication therapy for *H. pylori* infection is often challenging due to the increasing resistance of *H. pylori* against key antibiotics [8]. Of note, the eradication rate of standard triple therapy has rapidly fallen below an acceptable level in many parts of the world, with increasing clarithromycin resistance [9,10].

In line with this, current guidelines recommend bismuth quadruple therapy, containing bismuth combined with metronidazole and tetracycline, as the first-line therapy in regions with high levels of clarithromycin resistance or dual resistance [6]. Bismuth quadruple therapy is favored because it can overcome metronidazole resistance, with an acceptable eradication rate even in the presence of metronidazole resistance [9,11]. In addition, adding bismuth to antibiotics is considered advantageous due to its synergistic or additive effects [9]. However, studies on the synergistic effect of bismuth have shown inconsistent results, with some indicating no interactions [12]. Therefore, this study aims to address this knowledge gap by investigating whether a synergistic effect exists.

Bismuth is a heavy metal that has been used in medicines [13]. Bismuth salts have been used for diarrhea and peptic ulcers and have been discovered to offer anti-*H. pylori* properties [14,15,16]. Among the various bismuth preparations, bismuth subsalicylate (BSS) and colloidal bismuth subcitrate (CBS, De-Nol) are commonly used for *H. pylori* eradication therapy. However, despite the wide use of bismuth quadruple therapy, little research has investigated the in vitro susceptibility of *H. pylori* to bismuth compounds [17,18,19,20]. Moreover, only a small portion of bismuth’s mechanism of action on *H. pylori* is currently understood [13,21,22]. The aim of this study was to investigate the in vitro susceptibility of bismuth against *H. pylori*, with particular attention to the impact of pH using both reference *H. pylori* strains and clinically isolated *H. pylori* strains. Additionally, we evaluated whether bismuth has a synergistic effect with antibiotics that are frequently used for *H. pylori* eradication.

## 2. Results

### 2.1. Bismuth MICs for H. pylori Strains

**Figure 1:**
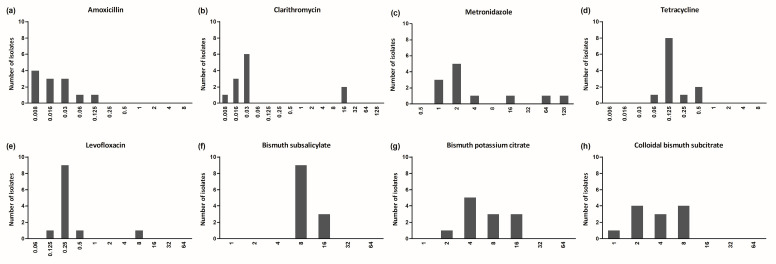
Distribution of minimum inhibitory concentrations for antibiotics and bismuth preparations against 12 *H. pylori* strains: (**a**) amoxicillin, (**b**) clarithromycin, (**c**) metronidazole, (**d**) tetracycline, (**e**) levofloxacin, (**f**) bismuth subsalicylate, (**g**) bismuth potassium citrate, and (**h**) colloidal bismuth subcitrate.

**Figure 2:**
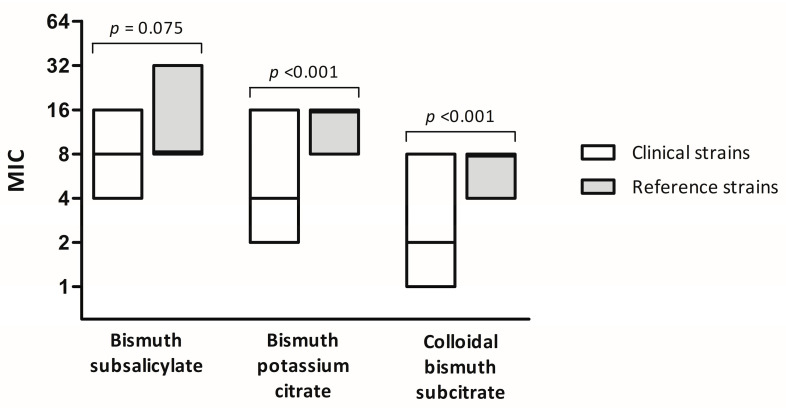
Comparison of minimum inhibitory concentrations for three bismuth preparations between clinical and reference *H. pylori* strains. The bar inside the box indicates the median. MIC, minimum inhibitory concentration.

The distributions of the MICs for all *H. pylori* strains for the three bismuth preparations and the five antibiotics are summarized in Table 1 and Figure 1. All strains were susceptible to amoxicillin and tetracycline, 16.7% were resistant to clarithromycin, and 25.0% were resistant to metronidazole. The MICs for BSS and bismuth potassium citrate ranged from 4 to 32 μg/mL and 2 to 16 μg/mL, respectively. For CBS, the median MIC value was 4 μg/mL (range: 1 to 8 μg/mL), and the MIC_50_ and MIC_90_ values were 4 μg/mL and 8 μg/mL, respectively. The MIC_90_ values of the bismuth preparations were higher than those of amoxicillin, tetracycline, and levofloxacin. In comparing the MICs between the reference and clinical *H. pylori* strains, the median MIC for BSS was 8 μg/mL for both the reference and clinical strains, while the MICs for bismuth potassium citrate and CBS were lower in the clinical strains than the reference strains, suggesting better activity (Figure 2).

The bismuth MICs for the reference strains at various pH values are listed in Table 2. The MICs among the three bismuth preparations were the lowest at pH 5.0 and similar through pH 6.0 to 8.0. In contrast, strain J99 showed a significantly lower MIC for CBS at pH 5.0 compared with at pH 7.0 (*p* = 0.003 for post hoc analysis).

### 2.2. Synergistic Effect Between Antibiotics and CBS

The results of the checkerboard assay for the three antibiotics (amoxicillin, clarithromycin, and metronidazole) and CBS are shown in Table 3 and Figure S1. Three reference strains, *H. pylori* 26695; J99; and ATCC 43504, which is a representative metronidazole-resistant strain; were tested. Although some MICs for each agent were one or two times lower than those of the individual agents when combined with another, all FIC index values were between 0.5 and 4, indicating no interaction between any of the antibiotics and CBS.

## 3. Discussion

In this study, we investigated the in vitro susceptibility of bismuth against *H. pylori* using an agar dilution technique. The bismuth preparations showed anti-*H. pylori* activity, with median MICs ranging from 4 to 16 μg/mL, in both the reference strains and clinically isolated *H. pylori* strains. Acidity did not alter the anti-bacterial activity of bismuth against *H. pylori* in reference strains. The potential synergistic effects of bismuth and antibiotics, including amoxicillin, clarithromycin, and metronidazole, were assessed using an agar dilution checkerboard assay, which revealed no interactions between bismuth and any of the antibiotics.

It is becoming increasingly apparent that bismuth plays an important role in *H. pylori* eradication therapy [23]. Based on previous studies, the MIC for bismuth has been reported to be between 4 and 32 μg/mL, which is consistent with our results [12,17,19,20]. In the present study, CBS showed a relatively lower MIC_90_ value than BSS or bismuth potassium citrate, suggesting better activity against *H. pylori*. The MICs of the bismuth preparations were higher than those of the antibiotics, indicating that the anti-bacterial activity of bismuth against *H. pylori* is much weaker than that of antibiotics. It is known that bismuth is not absorbed and exerts its activity through the local action of luminal bismuth, where concentrations greater than their MICs are expected [24]. However, there are few data available on the concentrations of bismuth in the mucus layer itself [21,25]. Moreover, the minimum or optimal bismuth dosage required for successful *H. pylori* eradication remains unclear, and bismuth-containing regimens have not yet been optimized.

Bismuth is a key component of a quadruple therapy consisting of a proton pump inhibitor, bismuth, metronidazole, and tetracycline, which is becoming an increasingly recommended alternative first-line treatment option for *H. pylori* eradication to standard triple therapy in areas with high clarithromycin resistance [6,7]. No resistance to bismuth has been reported so far, which may suggest the advantage of bismuth over antibiotics [21]. While bismuth alone can decrease bacterial load and rarely can clear an infection without concomitant antibiotic therapy, the addition of bismuth to eradication regimens increases the overall eradication rate of *H. pylori* [9,15,23]. The benefits of bismuth have been reported with various regimens for *H. pylori* eradication, and the improvement in the eradication rate was observed even in the presence of metronidazole resistance [23,26,27]. However, bismuth does not directly overcome antibiotic resistance, and the improved outcome with bismuth is additive [28].

Previous reports on the synergistic activity of bismuth with antibiotics were inconsistent, with bismuth appearing additive in some in vitro experiments and no interaction found in others [12,17,18,24,29]. In this study, synergistic effects between CBS and antibiotics were not observed. These results suggest that synergistic interactions may not be responsible for the clinical success of bismuth-containing regimens for *H. pylori* eradication. Alternatively, this may reflect a discrepancy between the in vitro and in vivo activities of bismuth and antibiotics against *H. pylori*. Indeed, a previous study found that bismuth subnitrate had a similar eradication efficacy in vivo as part of a triple therapy, with no in vitro activity against *H. pylori* [24]. The synergistic interactions observed in vitro did not guarantee any therapeutic benefit in vivo, and vice versa. A better understanding of the in vivo response of *H. pylori* to bismuth is critical for optimizing eradication therapy strategies.

Despite the wide use of bismuth quadruple therapy, bismuth is administered without a clear understanding of its mechanism of action. Several studies have highlighted interactions between proteins and enzymes, interference with metabolic pathways of iron, or direct effects on an organism, causing structural changes in the organism or loss of adherence to the epithelium [13,21,22,30,31,32,33,34]. Long-term incubation of bacteria with bismuth has been shown to alter their ultrastructure characteristics [21,35,36]. In a transcriptomic study, bismuth altered the expression levels of several iron metabolism-related proteins [34]. Moreover, it has been indicated that bismuth has a high affinity for proteins associated with nickel homeostasis [37,38]. While the exact mechanism of action of the anti-*H. pylori* activity associated with bismuth still needs to be investigated, much research suggests that bismuth interacts with cysteine residues or interferes with thiol-containing metal-binding sites in various target proteins of *H. pylori* [16,39,40].

A previous study found that CBS blocks protons from entering the bacterial cytoplasm rather than directly affecting urease or the urea channel, resulting in the maintenance of cytoplasmic pH within the range of increased metabolic activity of *H. pylori* in the setting of external acidification [41]. They suggested that this could be a dominant mechanism of action of CBS in acidic environments, allowing the enhanced efficacy of growth-dependent antibiotics or synergism with antibiotics. However, given that the bismuth quadruple therapy showed a reliable eradication rate without growth-dependent antibiotics, the mechanisms underlying the anti-bacterial effect of bismuth against *H. pylori* may be more complex and multifactorial. Further knowledge of the anti-bacterial effects of bismuth will lead to the optimization of bismuth-containing regimens for *H. pylori* eradication.

pH level often affects the bioavailability of drugs [42,43]. Studies have investigated the effect of pH changes on the activity of antibiotics used in the eradication therapy of *H. pylori* [44,45,46,47,48,49]. After the oral ingestion of bismuth compounds, bismuth salts are formed in the stomach, and these salts are taken up into the gastric mucus [14,21,50]. The solubility of bismuth compounds and their uptake into the gastric mucus also vary according to intragastric pH [20,21,24,25,32]. While previous studies have demonstrated the influence of pH on the effects of antibiotics against *H. pylori*, our study focused on the anti-*H. pylori* effects of bismuth under various pH conditions. The three bismuth preparations exhibited the lowest MIC values at pH 5; however, the differences between the various pH values were not statistically significant. These results suggest that acidity or intragastric pH may not be a major consideration when selecting bismuth for *H. pylori* eradication therapy. Since *H. pylori* does not live in the gastric lumen but rather in the gastric mucus, further studies are necessary to confirm our observations.

This study had several strengths. The agar dilution technique was used for susceptibility and synergy testing. Agar dilution is a reproducible and reliable method of testing antimicrobial susceptibility. The agar dilution method is preferred for broth dilution because it can be challenging to interpret growth inhibition in broth when there are colloidal suspensions of bismuth preparations. Second, the potential synergistic activity of bismuth and antibiotics was estimated using a checkerboard assay. Previous studies investigating the synergistic effects of bismuth and antibiotics were not based on established methodologies [18,24]. Using a checkerboard assay, the interaction between bismuth and antibiotics could be quantified and judged according to FIC indices. Finally, three types of bismuth preparations, BSS, bismuth potassium citrate, and CBS, were tested against both reference and clinical *H. pylori* strains to provide comprehensive data. Despite these strengths, this study had several limitations. First, the majority of investigations have used a colloidal suspension of bismuth in dimethyl sulfoxide due to the insoluble and precipitation-prone nature of bismuth preparations in a liquid medium. Second, the small number of clinically isolated *H. pylori* strains limited the generalizability of our results to a large cohort. Further studies with larger clinical samples are required to validate our findings. Additionally, the MIC values can vary according to the test method and the type of media or supplements used.

In conclusion, our study demonstrates its in vitro anti-bacterial activity against *H. pylori*. Although the role of bismuth in *H. pylori* eradication therapy requires further clarification, our findings highlight the potential benefits of bismuth-containing regimens in *H. pylori* eradication therapy and provide foundational insights into the anti-bacterial activity of bismuth against *H. pylori*. Considering escalating antibiotic resistance, incorporating bismuth into eradication regimens may offer a promising strategy to achieve *H. pylori* eradication while mitigating the risk of antibiotic resistance development. Further studies focused on optimizing bismuth use and elucidating its specific mechanisms will be critical for improving *H. pylori* eradication success rates.

## 4. Materials and Methods

### 4.1. Bacterial Strains

Three reference strains (*H. pylori* 26695, J99, and ATCC 43504) and nine clinically isolated *H. pylori* strains from patients with chronic atrophic gastritis were used. *H. pylori* strain 26695 was purchased from the American Type Culture Collection (ATCC 700392). Strains J99 and ATCC 43504 were kindly provided from Asan Medical Center, Seoul, Republic of Korea, and were cryopreserved in tryptic soy broth (Difco; Becton Dickinson and Company, Sparks, MD, USA) containing 15% of glycerol at −80 °C. To isolate the clinical *H. pylori* strains, mucosal biopsy specimens obtained from the antrum or corpus of the stomach during routine endoscopy were transported to the laboratory at Hallym University, Chuncheon, Republic of Korea, in sterile tubes containing normal saline within 4 h of endoscopy. The gastric biopsy specimens were processed and cultured on selective Brucella agar (Difco) plates supplemented with 7% defibrinated sheep blood (MBcell, Seoul, Republic of Korea), amphotericin B (Sigma-Aldrich, St Louis, MO, USA), and Campylobacter Skirrow’s supplement (MBcell) at 37 °C in a 10% CO_2_ incubator for 5 days. *H. pylori* was identified by colony morphology, a urease test, an oxidase test, a catalase test, and the PCR-detecting *glmM* gene using a forward primer sequence of 5′-AAG CTT TTA GGG GTG TTA GGG GTT T-3′ and reverse primer 5′-AAG CTT ACT TTC TAA CAC TAA CGC-3′ [51]. The isolates were then subcultured and maintained at −80 °C in tryptic soy broth with 15% glycerol until required for experiments.

### 4.2. Antimicrobial Susceptibility Testing

Antimicrobial susceptibility testing of *H. pylori* isolates was performed using the agar dilution technique with Mueller–Hinton agar (Difco) enriched with 5% defibrinated sheep blood and twofold dilutions of bismuth or antibiotics. Three formulations of bismuth, BSS (Sigma-Aldrich), bismuth potassium citrate (purchased from the procurement platform Aladdin), and CBS (De-Nol tablets; GC Biopharma Corp., Yongin, Republic of Korea) were powdered and placed into a colloidal suspension in dimethyl sulfoxide. Five antibiotics, namely, amoxicillin (Sigma-Aldrich), clarithromycin (Sigma-Aldrich), metronidazole (KisanBio, Seoul, Republic of Korea), tetracycline (Sigma-Aldrich), and levofloxacin (Sigma-Aldrich), were initially dissolved in appropriate buffer solutions or distilled water and further diluted twofold in distilled water. Each agent for the susceptibility test was poured and mixed with agar in 10 cm Petri dishes. The plates were prepared one day prior to inoculation.

All isolates were recovered from storage at −80 °C and cultured on Brucella agar plates enriched with 7% defibrinated sheep blood at 37 °C under microaerophilic conditions for 2–3 days. After subculturing, the bacterial suspensions were prepared in phosphate-buffered saline and adjusted to be equivalent to the 2.0 McFarland standard (approximately 6 × 10^8^ CFU/mL). The final inoculum of 5 μL per spot was directly inoculated onto the surface of each agar plate within 30 min of preparation. All plates were incubated at 37 °C under microaerophilic conditions and were examined visually after 72 h of incubation. Growth control plates without bismuth or antibiotics were also inoculated in each test series. The minimum inhibitory concentration (MIC) was defined as the lowest concentration that inhibited visible bacterial growth. The MIC_50_ and MIC_90_ values, representing the concentrations that inhibited 50% and 90% of the isolates, respectively, were calculated.

To investigate the influence of pH on the anti-bacterial effect of bismuth, the MICs were assessed under various pH conditions. In addition to the neutral pH, Mueller–Hinton agar plates with pH values of 5.0, 6.0, and 8.0, as measured with a surface pH meter, were prepared by adding HCl and NaOH, respectively, prior to sterilization. Suspensions of the three reference *H. pylori* strains were inoculated onto Mueller–Hinton agar plates containing BSS, bismuth potassium citrate, and CBS at each pH condition. All plates were incubated at 37 °C under microaerophilic atmosphere and were examined visually after 72 h.

There is no standardized breakpoint for *H. pylori* against bismuth preparations. The resistance breakpoints for amoxicillin, clarithromycin, metronidazole, tetracycline, and levofloxacin were 0.125 μg/mL, 0.25 μg/mL, 8 μg/mL, 1 μg/mL, and 1 μg/mL according to the EUCAST (European Committee on Antimicrobial Susceptibility Testing) breakpoint [52].

### 4.3. Checkerboard Assay for Synergy Test

The in vitro interactions between three antibiotics (amoxicillin, clarithromycin, and metronidazole) and CBS, a bismuth preparation only available with a prescription in South Korea, were quantified using a simplified agar dilution checkerboard assay [17]. The antibiotics and CBS were diluted horizontally and vertically in a geometric series, respectively, and each combination was mixed with Mueller–Hinton agar supplemented with 5% defibrinated sheep blood in 6-well plates. These concentrations were selected to reduce the number of agar plates while maintaining enough selectivity to identify the presence of synergistic or antagonistic interactions. After the inoculation of three reference *H. pylori* strains, all plates were incubated at 37 °C under microaerophilic conditions and were examined visually after 72 h of incubation.

To test whether there was a synergistic effect between the antibiotics and CBS, the fractional inhibitory concentration (FIC) indices were calculated using the formula FIC_AB_ index = FIC_A_ + FIC_B_ = (MIC_AB_/MIC_A_) + (MIC_BA_/MIC_B_), where MIC_A_ and MIC_B_ are the MICs of the two individual compounds and MIC_AB_ and MIC_BA_ represent the MICs of one compound when combined with the other. The combination was determined to be synergic when the FIC index was ≤0.5, have no interaction when the FIC index was >0.5 but ≤ 4, and be antagonistic when the FIC index was >4 [53]. All procedures were triplicated to confirm robustness.

### 4.4. Statistical Analysis

Proportions were used to summarize the descriptive statistics for the categorical variables, and medians and ranges were used to summarize those for the continuous variables. The bismuth MICs for the reference and clinical strains were compared using Student’s *t*-test. The bismuth MICs for the reference strains under various pH conditions were compared using ANOVA with a post hoc analysis. All the statistical analyses were performed using SPSS version 29.0 (IBM Corporation, Armonk, NY, USA). A *p*-value of <0.05 was considered significant, except for in the post hoc analysis, where a *p*-value of 0.05/6 = 0.008 (multiple testing for four categories) was considered statistically significant.

## Figures and Tables

**Table 1:** Minimum inhibitory concentrations for antibiotics and bismuth compounds against 12 *H. pylori* isolates, as determined by agar dilution methods.

	Inhibitory Concentration (μg/mL)			Resistance (%)	Breakpoint Criteria (μg/mL)
	Range	MIC_50_	MIC_90_		
Antibiotics					
Amoxicillin	0.004–0.125	0.016	0.06	0	>0.125
Clarithromycin	0.008–32	0.03	16	16.7	>0.25
Metronidazole	0.5–128	2	64	25.0	>8
Tetracycline	0.016–1	0.125	0.5	0	>1
Levofloxacin	0.125–8	0.25	0.5	8.3	>1
Bismuth					
Bismuth subsalicylate	4–32	8	16	NA	NA
Bismuth potassium citrate	2–16	4	16	NA	NA
Colloidal bismuth subcitrate	1–8	4	8	NA	NA

MIC, minimum inhibitory concentration; NA, not assessed.

**Table 2:** Minimum inhibitory concentrations for bismuth against three reference strains at various pH conditions.

	Minimum Inhibitory Concentration (μg/mL)				*p*-Value
	pH 5.0	pH 6.0	pH 7.0	pH 8.0	
Bismuth subsalicylate					
* H. pylori* strain 26695	2 (2–2)	8 (8–16)	8 (8–16)	8 (8–16)	0.337
J99	8 (4–8)	8 (8–16)	16 (8–16)	8 (8–16)	0.069
ATCC 43504	2 (1–4)	8 (4–16)	8 (8–32)	8 (8–16)	0.287
Bismuth potassium citrate					
* H. pylori* strain 26695	4 (2–8)	8 (4–8)	16 (8–16)	8 (8–8)	0.337
J99	4 (4–8)	8 (4–8)	16 (8–16)	8 (8–8)	0.038
ATCC 43504	4 (2–4)	8 (4–8)	16 (8–16)	8 (8–8)	0.011
Colloidal bismuth subcitrate					
*H. pylori* strain 26695	1 (1–2)	4 (4–4)	8 (4–8)	4 (4–4)	0.168
J99	4 (2–4) *	4 (4–4)	8 (4–8)	4 (4–8)	0.004
ATCC 43504	2 (1–4)	4 (4–8)	8 (4–8)	4 (4–8)	0.166

Data represent medians (ranges). * *p* < 0.008 in post hoc analysis (pH 5.0 vs. pH 7.0).

**Table 3:** Results of the agar dilution checkerboard assay for synergistic activity against bismuth compounds for three reference *H. pylori* strains.

Antibiotics Combined with Colloidal Bismuth Subcitrate	FIC Index Range		
	*H. pylori* 26695	J99	ATCC 43504
Amoxicillin	1.00–1.25	1.00–1.25	1.13–1.25
Clarithromycin	1.00–1.13	0.75–1.00	0.63–1.06
Metronidazole	0.63–0.75	0.56–1.00	0.50–1.01

FIC, fractional inhibitory concentration.

## Data Availability

The raw data supporting the conclusions of this article will be made available by the corresponding author on request.

## Supplementary Materials

The following supporting information can be downloaded at: https://www.mdpi.com/article/10.3390/antibiotics13111004/s1, Figure S1: Checkerboard assay for colloidal bismuth subcitrate (CBS) and antibiotics. Three reference strains, *H. pylori* 26695, J99, and ATCC 43504, were tested. White circles represent no growth, and grey circles represent growth of the organism. (**a**–**c**) Checkerboard assay for CBS and amoxicillin in *H. pylori* strain (**a**) 26695, (**b**) J99, and (**c**) ATCC 43504. (**d**–**f**) Checkerboard assay for CBS and clarithromycin for *H. pylori* strain (**d**) 26695, (**e**) J99, and (**f**) ATCC 43504. (**g**–**i**) Checkerboard assay for CBS and metronidazole in *H. pylori* strain (**g**) 26695, (**h**) J99, and (**i**) ATCC 43504.
