# Supplementary material for: In Vitro Susceptibility and Synergistic Effect of Bismuth Against Helicobacter pylori
Source: Antibiotics (Basel). 2024 Oct 25;13(11):1004. doi: 10.3390/antibiotics13111004 (PMC11591412; doi:10.3390/antibiotics13111004)
Supplement: Supplementary file 1 [file antibiotics-13-01004-s001.zip › antibiotics-3222258-supplementary.pdf]

Supplementary Information

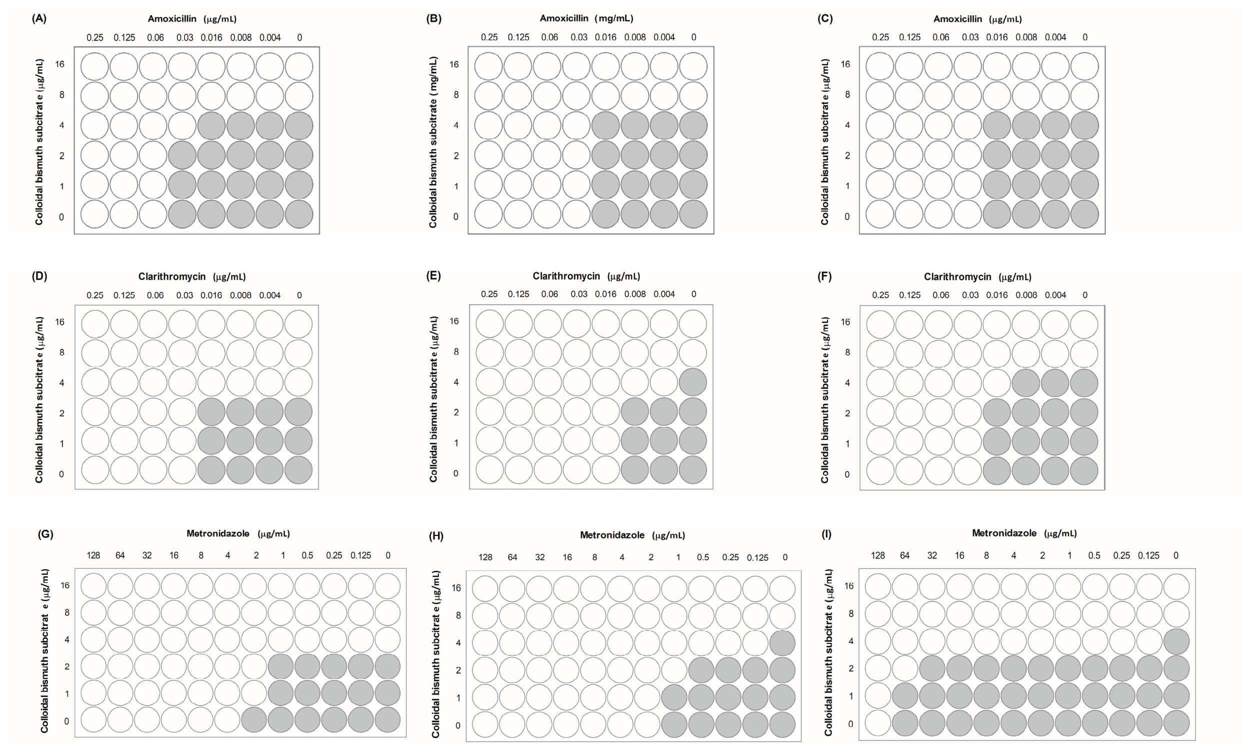

Figure S1. Checkerboard assay for colloidal bismuth subcitrate (CBS) and antibiotics. Three reference strains, *H. pylori* 26695, J99, and ATCC 43504, were tested. White circles represent no growth, and grey circles represent growth of the organism. (**A-C**) Checkerboard assay for CBS and amoxicillin in *H. pylori* strain (**A**) 26695, (**B**) J99, and (**C**) ATCC 43504. (**D-F**) Checkerboard assay for CBS and clarithromycin for *H. pylori* strain (**D**) 26695, (**E**) J99, and (**F**) ATCC 43504. (**G-I**) Checkerboard assay for CBS and metronidazole in *H. pylori* strain (**G**) 26695, (**H**) J99, and (**I**) ATCC 43504.
